# London's Great Problems

**Published:** 1890-12-13

**Authors:** 


### LONDON'S GREAT PROBLEM.

### NORTH KENSINGTON FRIENDLY WORKERS.

THE first annual meeting of the North Kensington Friendly Workers Amongst the Poor was held on Monday at All Saints' School-rooms, Kensington Park Road. Sir Roper Lethbridge, K.C.I.E., M.P., the honorary treasurer, occupied the chair, and was supported by Mr. Henry C. Burdett (chairman of the committee), Captain James, R.E., L.C.C., the Rev. Canon Trench (All Saints', Kensington Park), the Rev. J. Tuckwell (Westbourne Grove Chapel), the Rev. J. Stevens (Lancaster Road Congregational Church), Rev. R. Harris (Jewish Community), and others. It is now little more than a year ago since this scheme of friendly workers amongst the poor was first mooted, the outlines of it being given by the chairman at a meeting in August, 1889, at All Saints' School-room. The scheme was well received, and at a further meeting in November it was decided to try it experimentally in a prescribed district in North Kensington, selected as being a fairly typical one, Mr. Donald Mackenzie being appointed hon. secretary. In January and February of this year meetings were held to place the scheme before the public in the district, at which a committee was formed to carry the proposals into effect. This committee proceeded as soon as possible to ascertain the actual condition of the poor in their district, with the result that it has been found that the district contains 66 streets, of which 45 are poor or partly poor streets. Out of a total of 1,934 houses, 1,331 are to be found in these poor or partly poor streets, and 1,020 of them may be said to contain poor people. Of these 1,020 houses, as many as 820 have been already visited on behalf of the Committee, with the result that 1,187 families have given the Committee full particulars (which are now filed for confidential reference), and besides those recorded, there are in the 820 houses visited 217 other families who refuse information, as they feel themselves to be independent of extraneous aid. In the remaining 200 poor houses now being visited, it is estimated that there are about 300 families, and this visitation will be completed very shortly. Thus the Committee have to deal with 6,000 poor who reside within the district, the whole of which will soon have been visited, representing about 1,704 families, and of this number it appears that at present less than 200 persons are in immediate need of the Committee's assistance though it is probable that a much larger number of families will require help as the winter advances. In getting this information which has been achieved at the small cost of under £50, it will be seen that much has been accomplished, but this is really only a part of the work done, being the initial step, absolutely necessary, however, to the carrying on of the work. Besides this the Hon. Secretary has, during the year, dealt with a large number of cases of pressing need through the clergy and ministers of religion represented on the Committee, or through their congregations and friends through the existing organisations within the district. The last sentence touches the keynote of the Association's work. It does not seek, as all the speakers at the annual meeting clearly pointed out, to set up a new charitable society, but it seeks to bring all the charities already in existence in a given district into co-operation one with another, and so prevent the duplication of relief by the over-lapping of such charities. This is a purely undenominational work, and one in which all sects and creeds, and all bodies interested in the relief of the poor, as well as the local authorities; can and do join. That this is so is not now a matter of theory, but one of fact, for it has been clearly shown by the great interest and cordiality evinced by the ministers of all denominations—Church of England, Roman Catholics, and Nonconformists in their relations to one another on this, perhaps the only platform on which they can have no differences. All sects being agreed that this is a worthy, nay, an almost indispensable object, it surely should not be difficult for the association to obtain from the well-to-do residents within the area it covers the small sum, some £250 a-year, necessary for its maintenance, and to enable them to open a workroom, where women can be taught to sew, as well as be employed in sewing and needle-work; and to establish a labour bureau so that anyone requiring casual labour or work of any kind may obtain it on notifying their needs to the office, 28, Colville Square, where a list of respectable employés, men and women, will be kept.

